# The impact of dietary interventions on polycystic ovary syndrome patients with a BMI ≥25 kg/m^2^: A systematic review and meta‐analysis of randomized controlled trials

**DOI:** 10.1002/rmb2.12607

**Published:** 2024-09-30

**Authors:** Juan Yang, Jiahui Liang, Jinbang Xu, Tong Lin, Qiaoling Ye, Qiuping Lin, Feng Ji, Dan Shi

**Affiliations:** ^1^ Department of TCM Fujian Province Maternal and Child Health Care Hospital, Fujian Medical University Fuzhou China; ^2^ College of Acupuncture and Massage Fujian University of Traditional Chinese Medicine Fuzhou China

**Keywords:** BMI, calorie‐restricted diet, diet, low‐calorie combined low‐carbohydrate diet, polycystic ovary syndrome

## Abstract

**Background:**

Dietary interventions, recommended as a primary approach globally, benefit women with polycystic ovary syndrome (PCOS) by inducing weight loss and improving clinical symptoms, metabolism, and pregnancy results. However, the impact of diet on PCOS in individuals with BMI ≥ 25 kg/m^2^ is unclear. The aim of this review was to offer dietary guidance for these patients.

**Methods:**

Six databases, CNKI, Wanfang, VIP, PubMed, Cochrane Library, and Web of Science, were searched systematically from inception to December 2023 for clinical randomized controlled trials (RCT) on dietary interventions for PCOS. Two researchers independently screened and extracted data following pre‐defined inclusion criteria, with bias assessment using the Cochrane Handbook and Review Manager (version 5.4) software.

**Results:**

Nine RCTs with 559 participants were included. Among women with PCOS and obesity, compared to the control group, individuals who underwent dietary interventions experienced improvements in weight‐related Indicators, glycolipid metabolism, hormone‐related indicators, and fertility‐related outcomes. Subgroup analysis indicated that calorie‐restricted diets (CRDs) and low‐energy–low‐carb combined diets had advantages over other dietary interventions. Moreover, the overweight period was the optimal intervention period.

**Conclusions:**

Dietary interventions can improve the clinical manifestations of PCOS and pregnancy rates in patients with a BMI ≥ 25 kg/m^2^. Particularly, CRDs, low‐calorie–low‐carb combined diets, and low‐calorie–extract combined diets are recommended.

## Abstract 2

The study aimed to provide scientific recommendations for dietary interventions in PCOS patients with a BMI ≥ 25 kg/m^2^. It found that dietary intervention plays a positive role in weight management, improves clinical manifestations in PCOS patients with a BMI ≥ 25 kg/m^2^, and increases pregnancy rates. Among the recommended dietary interventions, an energy‐restricted diet, a low‐calorie combined with a low‐carb diet, and a low‐calorie combined with an extract diet are recommended for clinical application in a sequential order.

## 1 BACKGROUND

Polycystic ovary syndrome (PCOS) is the most common reproductive endocrine disorder and metabolic abnormality in women of reproductive age. The global prevalence of PCOS is 4%–21%, with overweight and obesity severely affecting 38%–88% of women with PCOS.^1^, ^2^ Currently, PCOS is characterized by an unclear etiology, a complex pathogenesis, and clinical heterogeneity, with chronic anovulation and hyperandrogenism as the main features. The clinical manifestations mainly include irregular menstrual cycles, hirsutism, acne, obesity, and infertility.^3^ Studies have shown that overweight PCOS patients often exhibit low gonadal hormone levels, insulin resistance, and high inflammation levels,^4^ and obesity can induce complications such as type 2 diabetes, cardiovascular disease, endometrial hyperplasia, and endometrial cancer in PCOS patients.^5^, ^6^, ^7^, ^8^, ^9^ In addition, epigenetic and environmental (diet, lifestyle) factors can influence the progression of PCOS, and obesity can induce clinical manifestations in genetically predisposed women and increase the incidence and prevalence of PCOS.^10^, ^11^ Therefore, it is meaningful to explore the impact of dietary interventions on PCOS patients from the perspective of overweight and obesity.

According to domestic and international guidelines, regardless of whether PCOS patients have fertility requirements, lifestyle adjustments should be made first to reduce weight to the normal range. Weight management is proposed as the primary intervention measure for PCOS patients. Lifestyle intervention, recommended as the first‐line treatment in the international evidence‐based guidelines for PCOS, mainly includes diet and exercise. Obesity is a common complication of PCOS, but there are still some patients who have a normal BMI and are lean. For lean PCOS patients with a normal BMI, there are no strict requirements for weight management. However, considering that lean PCOS patients also experience insulin resistance and similar health risks in other aspects as PCOS patients with obesity,^12^, ^13^ it is advisable to follow the general principles of a healthy diet. It is important to ensure a diverse and rich food intake in daily life along with regular exercise to increase the basal metabolic rate and improve endocrine abnormalities, irregular menstruation, and other issues.^14^ For PCOS patients with a BMI ≥25 kg/m^2^, obesity exacerbates clinical manifestations of PCOS‐related insulin resistance. Therefore, the range of different lifestyle interventions should be given more attention. In recent years, multiple systematic reviews and meta‐analyses have summarized the effects of exercise and diet on various clinical manifestations of PCOS patients. Among lifestyle interventions, exercise interventions can effectively reduce weight and related indicators such as body mass index (BMI) and waist circumference (WC), but more attention is given to cardiovascular metabolic risks and insulin resistance, with a lack of attention to reproductive‐related indicators.^15^, ^16^, ^17^ On the other hand, dietary interventions can effectively reduce weight, thereby alleviating symptoms such as menstrual abnormalities, hirsutism, and acne and improving insulin resistance and pregnancy outcomes.^18^, ^19^, ^20^ This is of great significance for discussing PCOS patients of childbearing age. However, there is currently no analysis of the impact of dietary interventions on overweight or obese PCOS patients, and the correlation between different dietary interventions and the weight, glucose and lipid metabolism, and pregnancy outcomes of PCOS patients with a BMI ≥25 kg/m^2^ is still unclear. This article aimed to address this uncertainty by conducting a meta‐analysis of the impact of different dietary interventions on relevant indicators in PCOS patients with a BMI ≥25 kg/m^2^ to better assist clinicians in guiding overweight or obese PCOS patients in choosing dietary plans.

## 2 MATERIALS AND METHODS

### 2.1 Search strategy

Two researchers conducted a comprehensive search of six databases, namely, the China National Knowledge Infrastructure (CNKI), Wanfang Data, VIP Chinese Technical Periodicals (VIP), PubMed, Web of Science, and Embase databases. The search covered the period from the inception of each database to December 2023. The search strategy primarily included the combination of subject terms and free words, and detailed searches were performed in the aforementioned databases. We employed various combinations of the following search terms: “polycystic ovary syndrome,” “PCOS,” “dietary intervention,” “Mediterranean diet,” “ketogenic diet,” “low GI diet,” “low carbohydrate diet,” and “hypertension dietary therapy.”

### 2.2 Study selection

Research that met the following inclusion criteria were included: (1) randomized controlled trials (RCTs); (2) studies including participants who met the World Health Organization's weight classification standards (overweight: BMI ≥ 25 kg/m^2^; obesity: BMI ≥ 30 kg/m^2^), met the Rotterdam criteria for PCOS as of 2003 and were between the ages of 18–45 years; (3) studies in which the intervention measures consisted of dietary therapy or dietary therapy combined with other treatments in the treatment group and general diet or other singular therapies in the control group; (4) studies including primary observation indicators such as weight‐related indicators (weight, BMI, WC, hip circumference [HC], and waist‐to‐hip ratio [WHR]), glucose metabolism indicators (fasting blood glucose [FBG], fasting serum insulin [FINS], and homeostatic model assessment of insulin resistance [HOMA‐IR]), lipid metabolism indicators (total cholesterol [TC], triglycerides [TGs], low‐density lipoprotein cholesterol [LDL‐C], and high‐density lipoprotein cholesterol [HDL‐C]), and hormone‐related indicators (follicle‐stimulating hormone [FSH], luteinizing hormone [LH], estrogen [E_2_], sex hormone‐binding globulin [SHBG], and dehydroepiandrosterone sulfate [DHEAS]) and secondary observation indicators such as pregnancy‐related indicators (number of successful ovulations and number of pregnancies).

The exclusion criteria were as follows: (1) non‐RCTs; (2) duplicate publications (studies with the same experimental subjects published by the same or different authors); (3) reviews, experiential studies, case reports, animal experiments, mechanism explorations, achievements, conferences, etc.; (4) studies in which the interventions involved a single dietary component (such as vitamins and calcium); and (5) studies with insufficient data and unreported primary outcomes.

### 2.3 Data extraction

Two independent reviewers (Jiahui Liang and Juan Yang) conducted literature screening and data extraction independently based on the inclusion and exclusion criteria. When there were conflicting opinions, they discussed with a third researcher. Two independent researchers (Dan Shi and Feng Ji) standardized the extraction of relevant data from the included literature, including basic study information (first author, publication year, study region, treatment period, sample size, and intervention measures), primary and secondary outcome measures, and relevant information for assessing the quality of the literature. The Cochrane risk‐of‐bias assessment tool was used for quality evaluation.

### 2.4 Risk of bias assessment

The quality and risk of bias of all the included literature in this study were assessed using the Cochrane Risk‐of‐Bias Assessment Tool. The contents evaluated by the Cochrane Risk‐of‐Bias Assessment Tool included the following: (1) random allocation methods; (2) concealed allocation; (3) blinding of research subjects and treatment implementers; (4) blinding of outcome assessors or peer reviewers; (5) completeness of outcome data; (6) selective reporting of study results; and (7) other sources of bias. The quality of the original studies was evaluated specifically using three levels of risk: low risk, high risk, and unknown risk. The final results are represented by a risk‐of‐bias diagram.

### 2.5 Data synthesis and analyses

In this study, the RevMan 5.4 software provided by the Cochrane Collaboration was used to perform statistical analysis of the results. *p* < 0.05 was considered to indicate statistical significance. Depending on the type of research report, the results are reported as follows: (1) for count data, the relative risk (RR) is used; (2) for continuous data, if all the studies reported using the same scale, the mean difference (MD) is used; if different methods or scales are reported, the standardized mean difference (SMD) is used. All the above are presented with 95% confidence intervals (CIs).

Due to the potential heterogeneity among the clinical studies, statistical heterogeneity was assessed by using the *Q*‐test (*p* value) and *I* square (*I* ^2^) statistics. When *p* > 0.05 and *I* ^2^ ≤ 50%, the data were considered homogeneous, and a fixed‐effect model was used for analysis. Conversely, when *p* < 0.05 and *I* ^2^ > 50%, high heterogeneity was considered, and a random‐effects model was employed for analysis. In cases of high heterogeneity, sensitivity analysis, and subgroup analysis were conducted to explore the potential sources of heterogeneity between studies. Sensitivity analysis involved the stepwise exclusion of studies to examine the robustness of the pooled estimates, while subgroup analysis was based on predefined factors such as diet type and BMI to conduct specific analyses, aiming to investigate the impact of diet therapy on overweight and/or obese PCOS patients. Since there were fewer than 10 trials included in the analysis, a funnel plot was not used to explore potential publication bias.

## 3 RESULTS

### 3.1 Study search and study characteristics

**FIGURE 1:**
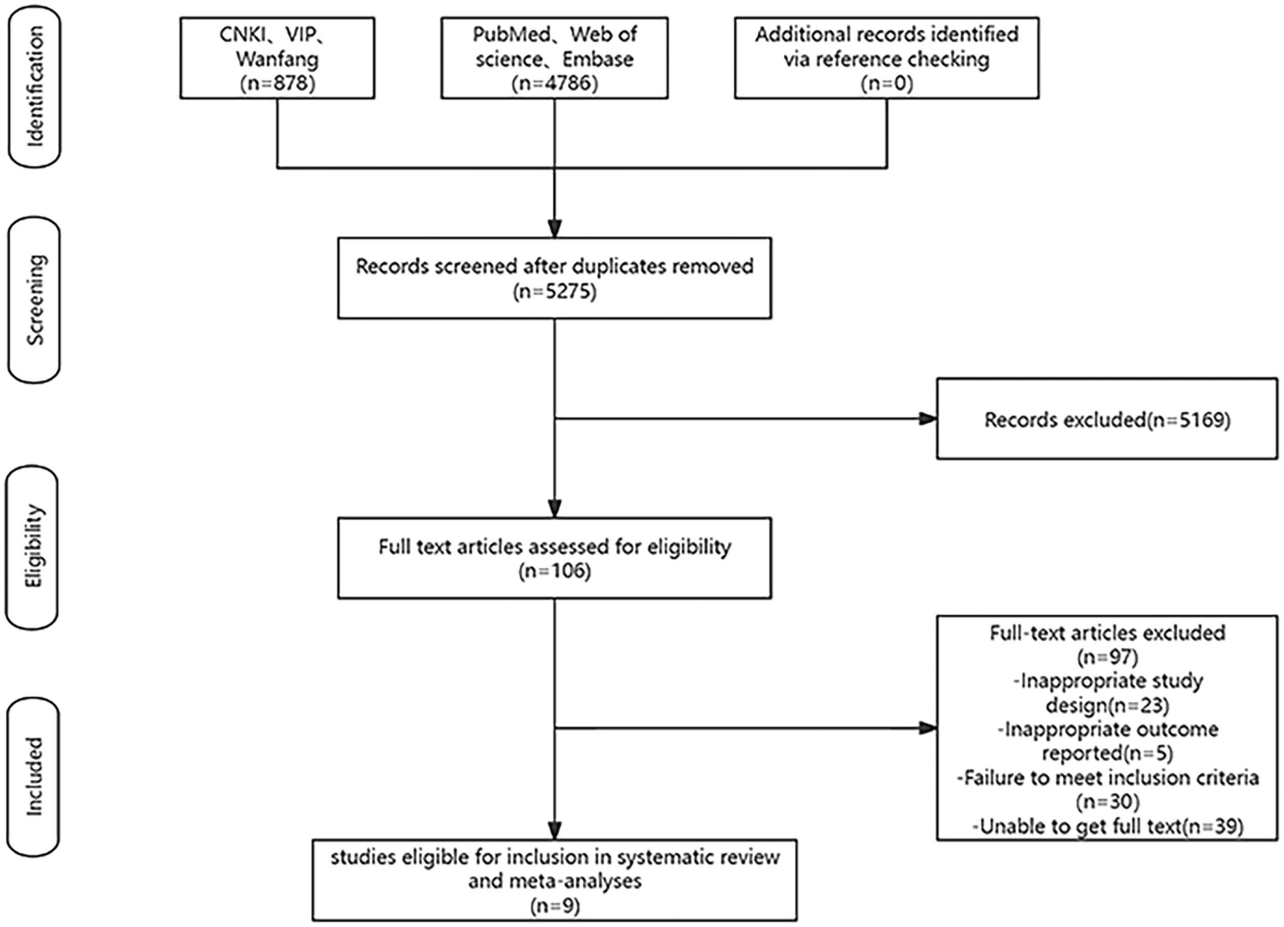
PRISMA flow diagram of study selection.

A preliminary literature search identified a total of 5664 articles, including 878 articles from the Chinese databases and 4786 articles from the foreign databases. After duplicate articles were removed using Endnote, 5275 articles remained. By reviewing the titles and abstracts and excluding articles did not meet the inclusion criteria, 5169 articles were excluded, and 106 articles remained. After thoroughly reading the full texts, 97 articles were further eliminated, resulting in the inclusion of 9 articles, all of which were published in journals. The literature search and screening process is shown in Figure 1.

The general characteristics of the studies included in this article are summarized as follows. The publication year of the studies ranged from 2015 to 2023, and the studies were conducted in China,^21^, ^22^, ^23^ Iran,^24^, ^25^, ^26^, ^27^ the United Kingdom,^28^ and Norway.^29^ The average age and BMI (kg/m^2^) of the female participants in the intervention and control groups were 27.5 to 33.81 years and 27.41 to 44 kg/m^2^, respectively. A total of 559 study participants were included in the 9 studies, with 284 participants in the treatment group and 275 participants in the control group. The intervention measures in the treatment groups include the Mediterranean diet in one study,^21^ a restricted‐energy diet in one study,^22^ a low‐calorie combined with a low‐carbohydrate diet in one study,^23^ a low‐calorie‐extract combined diets in three studies,^24^, ^25^, ^29^ a low‐calorie and low‐carbohydrate diet combined with an extract diet in one study,^26^ a very low‐calorie diet in one study,^28^ and a low‐carbohydrate combined with an extract diet in one study.^27^ The intervention measures in the control groups include Western medicine in two studies,^22^, ^23^ general dietary guidance in one study,^21^ an intervention plus placebo in five studies,^24^, ^25^, ^26^, ^27^, ^29^ and a moderate energy‐deficient diet in one study.^28^ The basic information of the included studies, such as the disease duration, participant age, specific intervention measures, and outcome indicators, are shown in Table 1.

**TABLE 1:** General characteristics of the included studies.

Author/year	PCOS criteria	Methods		Sample size		Age (years)		BMI (kg/m^2^)		Cycles (months)	Outcomes
		Treatment	Control	Treatment	Control	Treatment	Control	Treatment	Control		
Li X 2017	Rotterdam 2003	Mediterranean diet	General diet	20	20	28.65 ± 3.28	27.85 ± 3.27	27.41 ± 1.24	27.85 ± 2.17	3	Weight, BMI, LH, the number of pregnancies
Jinhui W 2020	Rotterdam 2003	Calorie‐restricted diet	Metformin tablets	38	38	29.07 ± 5.29	28.91 ± 5.52	29.51 ± 1.80	29.40 ± 2.01	3	BMI, FSH, LH, E_2_, FBG, FINS, HOMA‐IR, HDL‐C, LDL‐C, TG, TC, the number of pregnancies, the number of ovulations
Yanli Y 2018	Rotterdam 2003	Low‐carbohydrate, low‐energy diets, daying‐35	Daying‐35	40	30	28.06 ± 4.21	28.06 ± 4.21	28.58 ± 1.42	28.69 ± 1.36	3	BMI, FSH, LH, E_2_, FBG, FINS, HOMA‐IR, the number of pregnancies, the number of ovulations
Cheshmeh S 2022	Rotterdam 2003	Three 1000 mg green cardamom capsules, low‐calorie diet	Three 1000 mg starch powder capsules, low‐calorie diet	99	95	32.99 ± 5.57	33.81 ± 5.42	34.78 ± 3.39	35.18 ± 5.16	4	Weight, BMI, FSH, LH, DHEA, SHBG
Tabrizi F 2020	Rotterdam 2003	5 g/day thylakoid‐rich spinach extract, low‐calorie diet	5 g cornstarch, low‐calorie diet	21	23	31.86 ± 2.35	32.04 ± 2.83	35.13 ± 2.16	35.31 ± 2.77	3	Weight, BMI, WC, WHR, FSH, LH, DHEA, SHBG, FAI, FBG, FINS, HOMA‐IR
Nadjarzadeh A 2021	Rotterdam 2003	Hypocaloric high‐protein diet + fennel (2 capsule/day)	Hypocaloric high‐protein diet + placebo (HHPP)	16	16	27.50 ± 7.97	29.43 ± 6.60	31.25 ± 3.02	32.8 ± 5.34	3	Weight, BMI, WC, HC, WHR, SHBG, FAI
Deshmukh H 2023	Rotterdam 2003	Very‐low‐calorie diet	Conventional energy deficit	11	11	27.7 ± 3.8	28.1 ± 5.6	37.8 ± 3.9	37.6 ± 5.07	4	Weight, BMI, WC, WHR, FSH, LH, DHEA, SHBG, FAI, FBG, TC, TG
Marnani, EH 2020	Rotterdam 2003	High‐protein, low‐carbohydrate diet + fennel capsule (HPF)	High‐protein, low‐carbohydrate diet + placebo capsule (HPP)	15	15	28.06 ± 7.91	29.86 ± 6.59	31.35 ± 3.10	34.48 ± 5.66	3	FBG, FINS, HOMA‐IR,
Johnson L K 2015	Rotterdam 2003	Low‐fructose, low‐calorie diet	A whole‐grain crispbread (CB), low‐calorie diet	24	27	29.0 ± 6.3	29.0 ± 5.6	43.0 + 5.6	44.0 ± 5.8	2	Weight, BMI, WC, HC, FSH, LH, E_2_, SHBG, HOMA‐IR, TC, TG, LDL‐C, HDL‐C

Abbreviations: BMI, Body Mass Index; DHEA, dehydroepiandrosterone; E_2_, estrogenic hormone; FAI, free androgen index; FSH, follicle‐stimulating hormone; HC, hip circumference; HDL‐C, high density lipoprotein cholesterol; HOMA‐IR, homeostasis model assessment of insulin resistance; LDL‐C, low‐density lipoprotein cholesterol; LH, luteinizing hormone; PCOS, polycystic ovary syndrome; SHBG, sex hormone binding globulin; TC, total cholesterol; TG, triglyceride; WC, waist circumference; WHR, waist‐to‐hip ratio.

### 3.2 Risk of bias assessment

**FIGURE 2:**
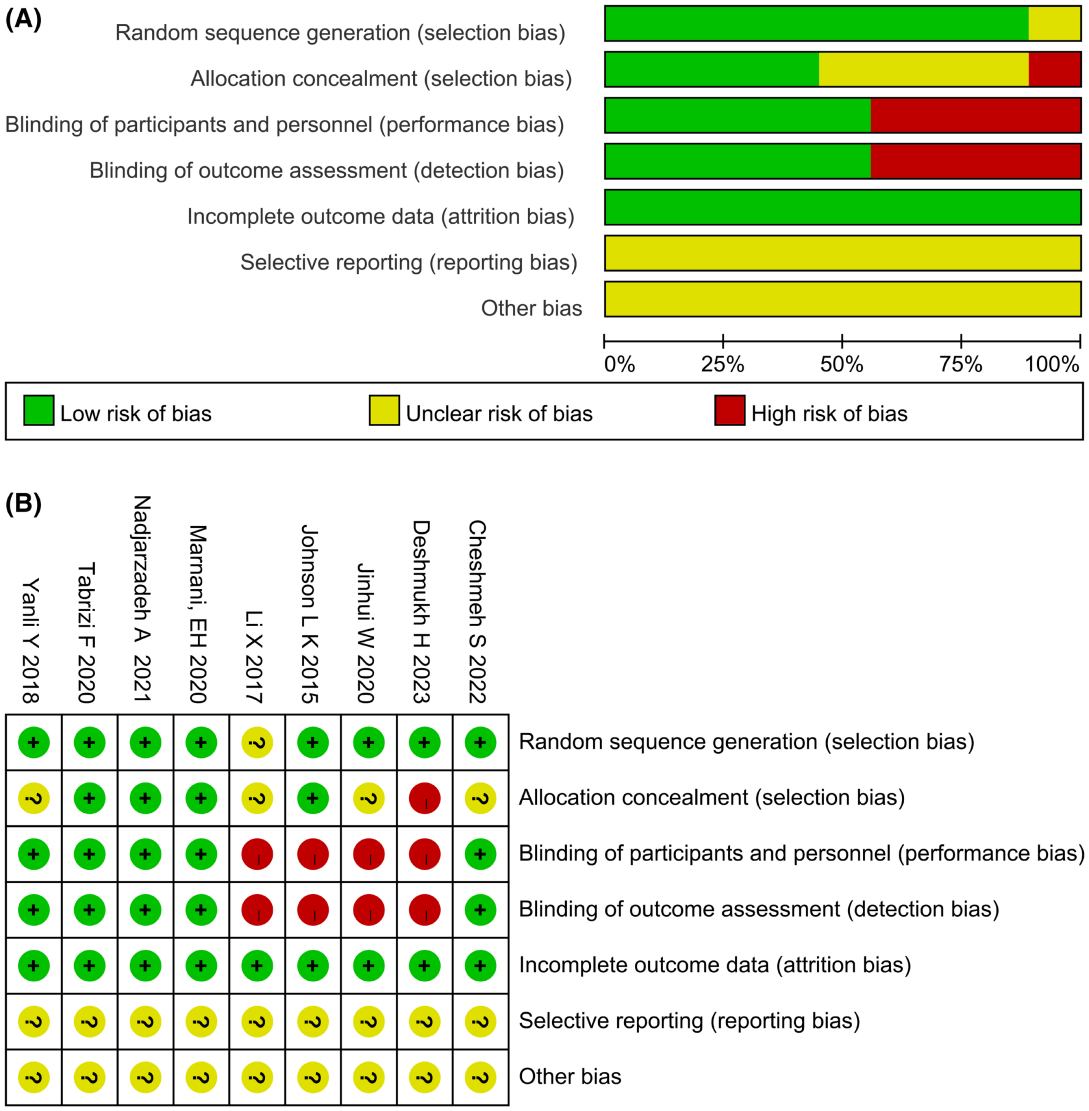
Assessments about risk‐of‐bias of included studies. (A) risk of bias graph and (B) risk of bias summary.

Regarding the randomization aspect, eight studies^21^, ^22^, ^23^, ^24^, ^25^, ^26^, ^27^, ^29^ used randomization for grouping and were evaluated as having a low risk of bias; one study^28^ did not provide detailed information on the specific randomization method and was evaluated as having an unknown risk of bias. Regarding the blinding aspect, four studies^25^, ^26^, ^27^, ^29^ mentioned specific blinding and were evaluated as having a low risk of bias, while one study^28^ explicitly stated that an open randomization scheme without blinding was used and was evaluated as having a high risk of bias. The remaining four studies^21^, ^22^, ^23^, ^24^ did not report the use of a blinding scheme and were evaluated as having an unknown risk of bias. Regarding blinding of the study participants, treatment implementers, and outcome assessors, five studies^23^, ^24^, ^25^, ^26^, ^27^ implemented double‐blinding and were evaluated as having a low risk of bias, while four articles^21^, ^22^, ^28^, ^29^ did not implement blinding and were evaluated as having a high risk of bias. Due to the completeness of the outcome data, all nine studies^21^, ^22^, ^23^, ^24^, ^25^, ^26^, ^27^, ^28^, ^29^ did not have any dropouts or missing data and were evaluated as having a low risk of bias. Regarding the selective reporting of study results, all nine studies^21^, ^22^, ^23^, ^24^, ^25^, ^26^, ^27^, ^28^, ^29^ did not provide comprehensive reports and were evaluated as having an unknown risk of bias. Regarding other biases, all nine studies^21^, ^22^, ^23^, ^24^, ^25^, ^26^, ^27^, ^28^, ^29^ did not provide information on factors that could potentially affect the results and were evaluated as having an unknown risk of bias. The quality assessment results are shown in Figure 2.

### 3.3 Synthesis of results

#### 3.3.1 Weight‐related indicators

**FIGURE 3:**
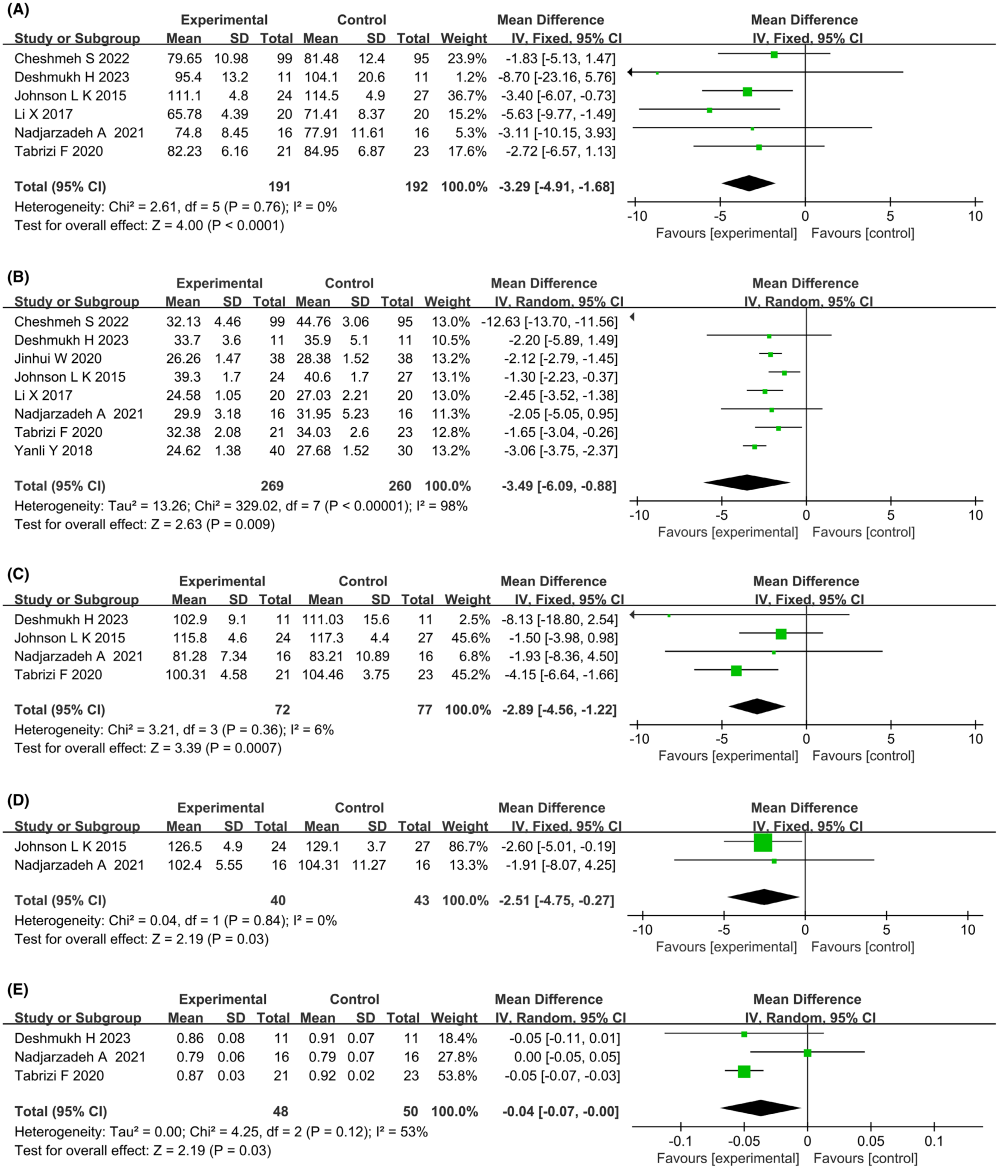
Forest plots of meta‐analysis for (A) weight, (B) body mass index (BMI), (C) waist circumference (WC), (D) hip circumference (HC), (E) waist‐to‐hip ratio (WHR). CI, confidence interval; df, degrees of freedom; IV, inverse variance; SD, standard deviation.

A total of eight studies reported the effects of dietary interventions on weight‐related indicators. Heterogeneity tests showed high heterogeneity for BMI (*p* < 0.00001, *I* ^2^ = 98%) and the WHR (*p* = 0.12, *I* ^2^ = 53%), while the other indicators were determined to be homogeneous using a random effects model. Meta‐analysis showed that six studies^21^, ^24^, ^25^, ^26^, ^28^, ^29^ reported weight values (MD: −3.29; 95% CI: −4.91, −1.68; *p* < 0.0001) (Figure 3A), eight studies^21^, ^22^, ^23^, ^24^, ^25^, ^26^, ^28^, ^29^ reported BMI values (MD: −3.49; 95% CI: −6.09, −0.88; *p* = 0.009) (Figure 3B), four studies^25^, ^26^, ^28^, ^29^ reported WC values (MD: −2.89; 95% CI: −4.56, −1.22; *p* = 0.0007) (Figure 3C), two studies^26^, ^29^ reported HC values (MD: −2.51; 95% CI: −4.75, −0.27; *p* = 0.03) (Figure 3D), and three studies^25^, ^26^, ^28^ reported WHR values (MD: −0.04; 95% CI: −0.07, 0.00; *p* = 0.03) (Figure 3E). Compared to the control group, the dietary intervention group showed improvements in terms of weight, BMI, WC, HC, and WHR. Subgroup analysis showed that the Mediterranean diet group had improvements in weight and BMI, the calorie‐restriction diet combined with a low‐carbohydrate diet group had improvements in BMI, the calorie‐restriction diet combined with an extract diet group had improvements in weight, WC, and the WHR. Furthermore, overweight and obese PCOS patients in the dietary intervention group showed improvements in weight. Subgroup analysis results for weight‐related indicators are shown in Table 2.

**TABLE 2:** Subgroup analyses evaluated the effects of different dietary regimens and BMI types on weight‐related indicators in overweight/obese patients with PCOS.

Subgrouped by	Number of trials	Effect size	95% CI	*p* value for effect estimates^2^	*I* ^*2*^, %	*p* value for within‐subgroup heterogeneity^2^	*p* value for between‐subgroup heterogeneity^3^
Weight, kg							
Dietary patterns							
Mediterranean diet	1	−5.63	−9.77 to −1.49			0.008	<0.0001
Low‐calorie combined extract diet	3	−2.77	−4.59 to −0.94	0.77	0	0.003	
Low‐calorie, low‐carbohydrate combined extract diet	1	−3.11	−10.15 to 3.93			0.39	
Very‐low‐calorie diet	1	−8.70	−23.16 to 5.76			0.24	
BMI types							
Overweight (≥25 kg/m^2^)	1	−5.63	−9.77 to −1.49				<0.0001
Obese (≥30 kg/m^2^)	5	−2.88	−4.63 to −1.12	0.88	0	0.001	
BMI, kg/m^2^							
Dietary patterns							
Mediterranean diet	1	−2.45	−3.52 to −1.38			<0.00001	<0.00001
Calorie‐restricted diet	1	−2.12	−2.79 to −1.45			<0.00001	
Low‐energy and low‐carbohydrate diet	1	−3.06	−3.75 to −2.37			<0.00001	
Low‐calorie combined extract diet	3	−1.41	−2.18 to −0.63	0.68	0	0.0004	
Low‐calorie, low‐carbohydrate combined extract diet	1	−2.05	−5.05 to 0.95			0.18	
Very‐low‐calorie diet	1	−2.20	−5.89 to 1.49			0.24	
Waist circumference, cm							
Dietary patterns							
Low‐calorie combined extract diet	2	−2.82	−4.58 to −1.06	0.14	54	0.002	0.0007
Low‐calorie, low‐carbohydrate combined extract diet	1	−1.93	−8.36 to 4.50			0.56	
Very‐low‐calorie diet	1	−8.13	−18.80 to 2.54			0.14	
Hip circumference, cm							
Dietary patterns							
Low‐calorie combined extract diet	1	−2.60	−5.01 to −0.19			0.03	0.03
Low‐calorie, low‐carbohydrate combined extract diet	1	−1.91	−8.07 to 4.25			0.54	
Waist‐to‐hip ratio							
Dietary patterns							
Low‐calorie combined extract diet	1	−0.05	−0.07 to −0.03			<0.00001	<0.00001
Low‐calorie, low‐carbohydrate combined extract diet	1	0.00	−0.05 to 0.05			1.00	
Very‐low‐calorie diet	1	−0.05	−0.11 to 0.01			0.12	

#### 3.3.2 Lipid metabolism

**FIGURE 4:**
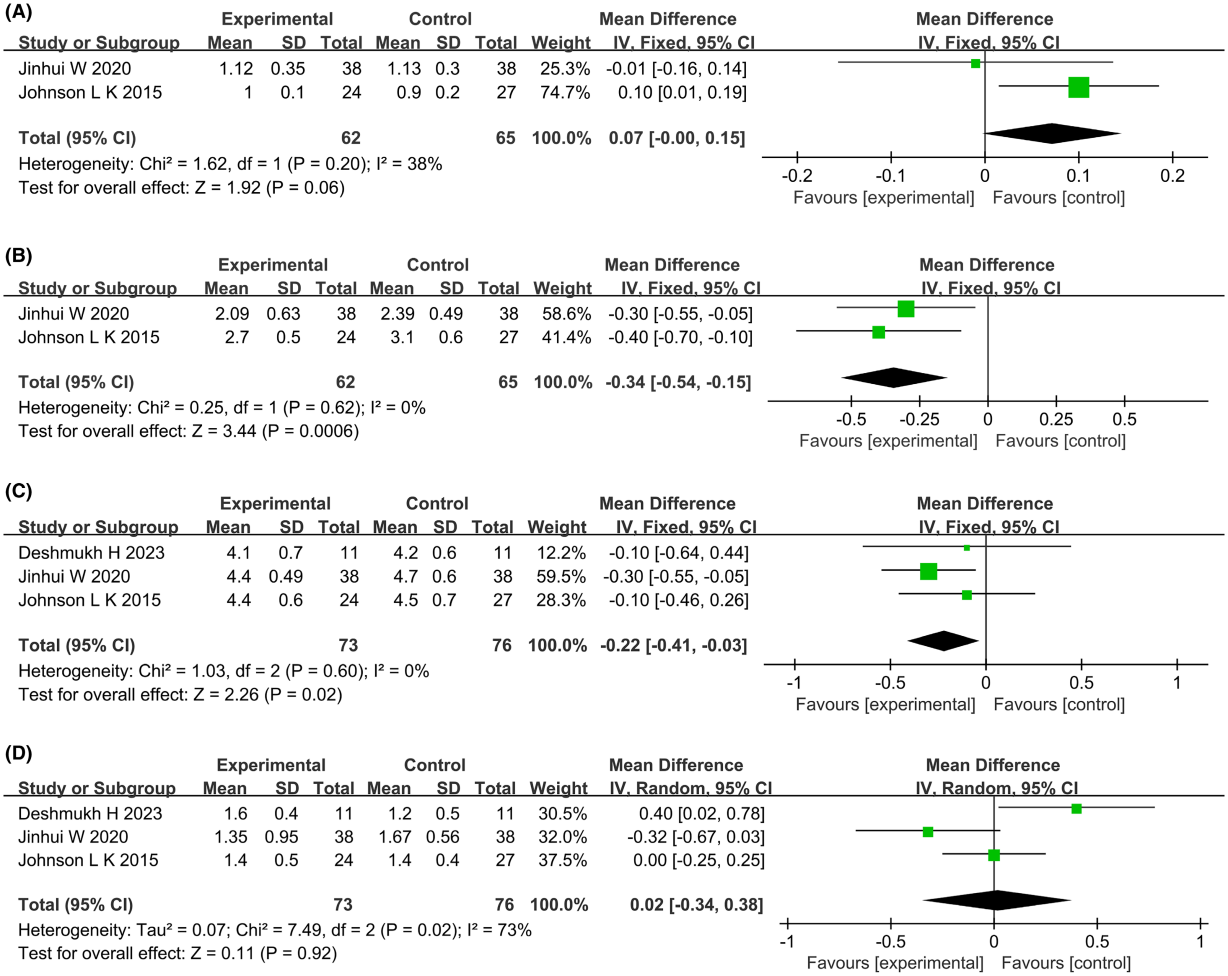
Forest plots of meta‐analysis for (A) high density lipoprotein cholesterol, (HDL‐C), (B) low‐density lipoprotein‐cholesterol (LDL‐C), (C) total cholesterol (TC), (D) triglyceride (TG). CI, confidence interval; df, degrees of freedom; IV, inverse variance; SD, standard deviation.

Three studies reported the effects of dietary interventions on lipid metabolism. Heterogeneity tests showed high heterogeneity in TG level (*p* = 0.02, *I* ^2^ = 73%), while the remaining indicators showed homogeneity and were analyzed using fixed‐effect models. Meta‐analysis showed that two studies^22^, ^29^ reported HDL‐C levels (MD: 0.07; 95% CI: −0.00, 0.15; *p* = 0.06) (Figure 4A), two studies^22^, ^29^ reported LDL‐C levels (MD: −0.34; 95% CI: −0.54, −0.15; *p* = 0.0006) (Figure 4B), three studies^22^, ^28^, ^29^ reported TC levels (MD: −0.22; 95% CI: −0.41, −0.03; *p* = 0.02) (Figure 4C), and three studies^22^, ^28^, ^29^ reported TG levels (MD: 0.02; 95% CI: −0.34, 0.38; *p* = 0.92) (Figure 4D). Compared to the control group, the dietary intervention group showed improvements in LDL‐C and TC levels. Subgroup analysis showed that the energy‐restricted diet group had improved TC and LDL‐C levels, and the low‐calorie combined with an extract diet group had improved LDL‐C levels. Additionally, overweight PCOS patients in the dietary intervention group showed improved TC levels. Subgroup analysis results for lipid metabolism are shown in Table 3.

**TABLE 3:** Subgroup analyses evaluated the effects of different dietary regimens and BMI types on lipid metabolism in overweight/obese patients with PCOS.

Subgrouped by	Number of trials	Effect size	95% CI	*p* value for effect estimates^2^	*I* ^*2*^, %	*p* value for within‐subgroup heterogeneity^2^	*p* value for between‐subgroup heterogeneity^3^
LDL‐C, mmol/L							
Dietary patterns							
Calorie‐restricted diet	1	−0.30	−0.55 to −0.05			0.02	0.0006
Low‐calorie combined extract diet	1	−0.40	−0.70 to −0.10			0.009	
BMI types							
Overweight (≥25 kg/m^2^)	1	−0.30	−0.55 to −0.05			0.02	0.0006
Obese (≥30 kg/m^2^)	1	−0.40	−0.70 to −0.10			0.009	
TC, mmol/L							
Dietary patterns							
Calorie‐restricted diet	1	−0.30	−0.55 to −0.05			0.02	0.02
Very‐low‐calorie diet	1	−0.10	−0.64 to 0.44			0.72	
Low‐calorie combined extract diet	1	−0.40	−0.70 to −0.10			0.58	
BMI types							
Overweight (≥25 kg/m^2^)	1	−0.30	−0.55 to −0.05			0.02	0.02
Obese (≥30 kg/m^2^)	2	−0.10	−0.40 to 0.20	1	0	0.51	

#### 3.3.3 Carbohydrate metabolism

**FIGURE 5:**
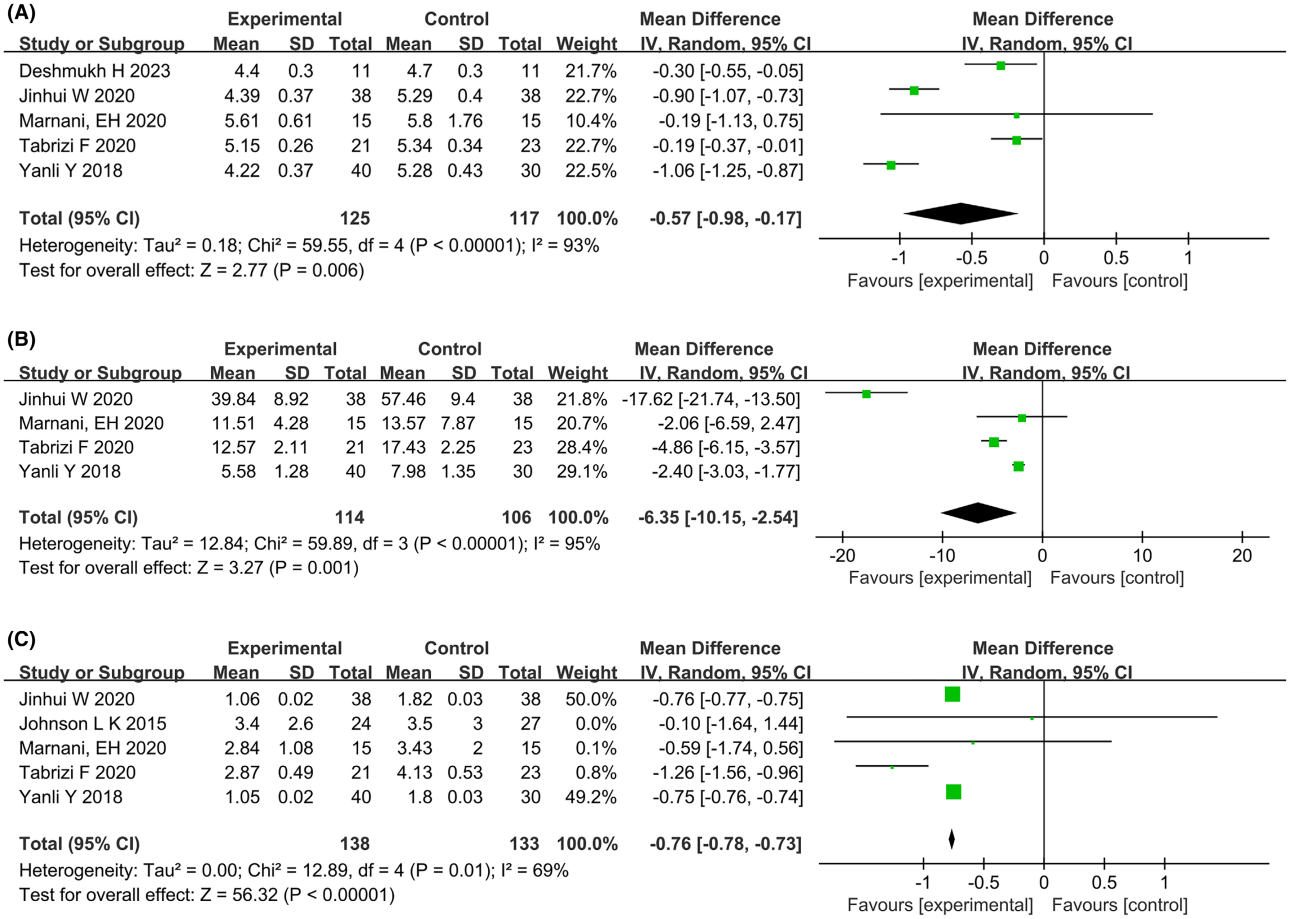
Forest plots of meta‐analysis for (A) fasting blood glucose (FBG), (B) fasting serum insulin (FINS), (C) homeostatic model assessment of insulin resistance (HOMA‐IR). CI, confidence interval; df, degrees of freedom; IV, inverse variance; SD, standard deviation.

Five studies reported the effects of dietary interventions on glucose metabolism. Heterogeneity tests showed high heterogeneity in FBG levels (*p* < 0.00001, *I* ^2^ = 93%), FINS levels (*p* < 0.00001, *I* ^2^ = 95%), and HOMA‐IR values (*p* = 0.01, *I* ^2^ = 69%), all using a random‐effects model. Meta‐analysis showed that five studies^22^, ^23^, ^25^, ^27^, ^28^ reported FBG levels (MD: −0.57; 95% CI: −0.98, −0.17; *p* = 0.006) (Figure 5A), four studies^22^, ^23^, ^25^, ^27^ reported FINS levels (MD: −6.35; 95% CI: −10.15, −2.54; *p* = 0.001) (Figure 5B), and five studies^22^, ^23^, ^25^, ^27^, ^29^ reported HOMA‐IR values (MD: −0.76; 95% CI: −0.78, −0.73; *p* < 0.00001) (Figure 5C). The dietary intervention group showed improved FBG levels, FINS levels, and HOMA‐IR values compared to the control group. Subgroup analysis showed that the energy‐restricted diet combined with a low‐carbohydrate diet group had improved FBG levels, FINS levels, and HOMA‐IR values; the low‐calorie diet combined with an extract diet group had improved FBG and FINS levels; and the very low‐calorie diet group had improved FBG levels. The dietary intervention group also showed improved FINS levels and HOMA‐IR values in overweight PCOS patients and HOMA‐IR values in obese PCOS patients. Subgroup analysis results for carbohydrate metabolism are shown in Table 4.

**TABLE 4:** Subgroup analyses evaluated the effects of different dietary regimens and BMI types on carbohydrate metabolism in overweight/obese patients with PCOS.

Subgrouped by	Number of trials	Effect size	95% CI	*p* value for effect estimates^2^	*I* ^*2*^, %	*p* value for within‐subgroup heterogeneity^2^	*p* value for between‐subgroup heterogeneity^3^
FBG, mmol/L							
Dietary patterns							
Calorie‐restricted diet	1	−0.90	−1.07 to −0.73			<0.00001	0.006
Low‐energy and low‐carbohydrate diet	1	−1.06	−1.25 to −0.87			<0.00001	
Low‐calorie combined extract diet	1	−0.19	−0.37 to −0.01			0.04	
Low carbohydrate combined extract diet	1	−0.19	−1.13 to 0.75			0.69	
Very‐low‐calorie diet	1	−0.30	−0.55 to −0.05			0.02	
BMI types							
Overweight (≥25 kg/m^2^)	2	−0.97	−1.13 to −0.82	0.23	32	<0.00001	0.006
Obese (≥30 kg/m^2^)	3	−0.23	−0.37 to −0.08	0.78	0	0.002	
FINS, mU/L							
Dietary patterns							
Calorie‐restricted diet	1	−17.62	−21.74 to −13.50			<0.00001	0.001
Low‐energy and low‐carbohydrate diet	1	−2.40	−3.03 to −1.77			<0.00001	
Low‐calorie combined extract diet	1	−4.86	−6.15 to −3.57			<0.00001	
Low carbohydrate combined extract diet	1	−2.06	−6.59 to 2.47			0.37	
BMI types							
Overweight (≥25 kg/m^2^)	2	−9.87	−24.78 to 5.04	<0.00001	98	0.19	0.001
Obese (≥30 kg/m^2^)	2	−4.34	−6.48 to −2.20	0.24	26	<0.0001	
HOMA‐IR							
Dietary patterns							
Calorie‐restricted diet	1	−0.76	−0.77 to −0.75			<0.00001	<0.00001
Low‐energy and low‐carbohydrate diet	1	−0.75	−0.76 to −0.74			<0.00001	
Low‐calorie combined extract diet	2	−0.93	−1.96 to 0.09	0.15	53	0.07	
Low carbohydrate combined extract diet	1	−0.59	−1.74 to 0.56			0.31	
BMI type							
Overweight (≥25 kg/m^2^)	2	−0.76	−0.76 to −0.75	0.25	26	<0.00001	<0.00001
Obese (≥30 kg/m^2^)	3	−1.18	−1.46 to −0.89	0.20	37	<0.00001	

#### 3.3.4 Hormones and related indicators

**FIGURE 6:**
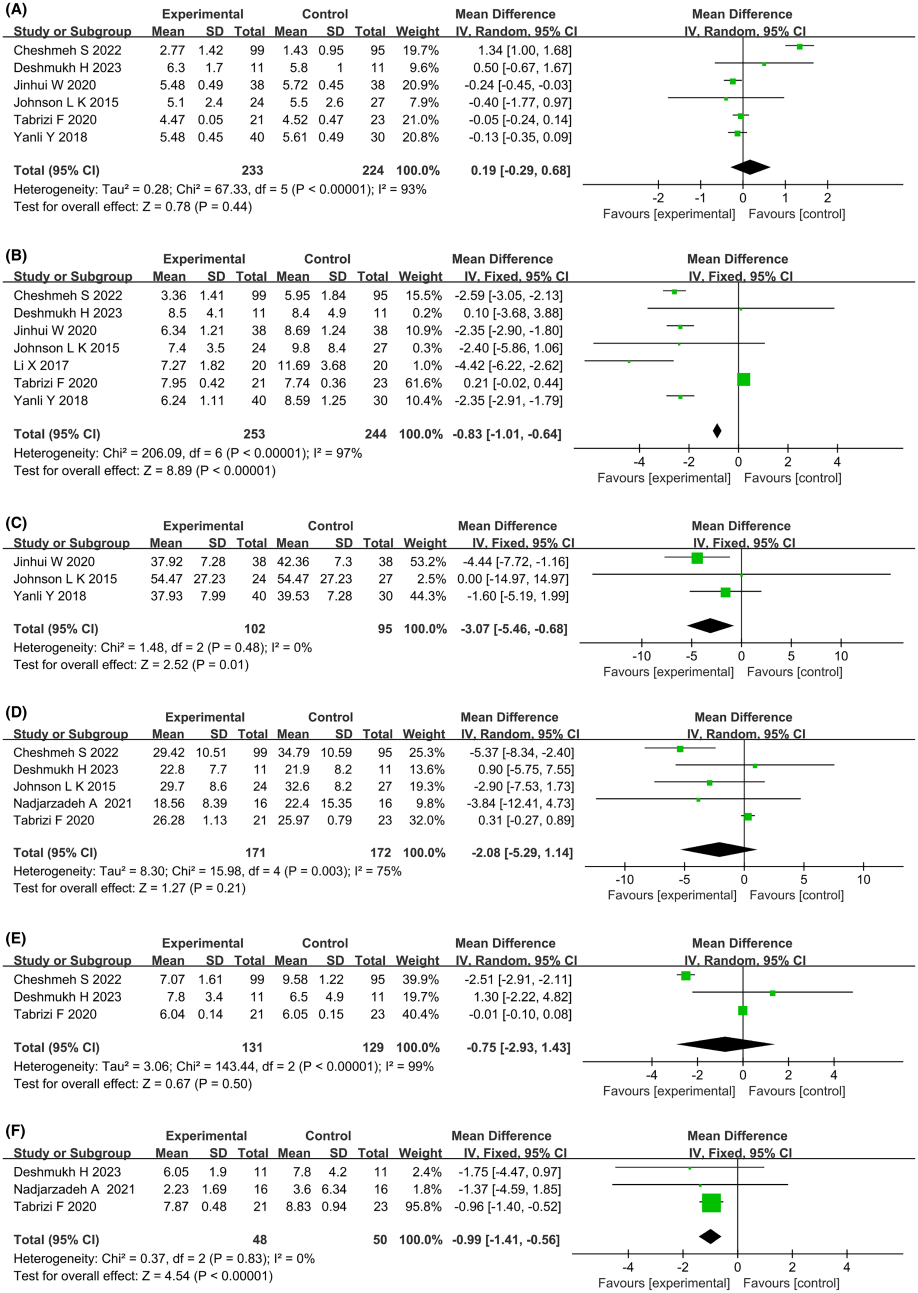
Forest plots of meta‐analysis for (A) follicle‐stimulating hormone (FSH), (B) luteinizing hormone (LH), (C) estradiol (E_2_), (D) sex hormone‐binding globulin (SHBG), (E) dehydroepiandrosterone (DHEA), (F) free androgen index (FAI). CI, confidence interval; df, degrees of freedom; IV, inverse variance; SD, standard deviation.

Eight studies reported the effects of dietary interventions on hormones and related indicators. Heterogeneity tests showed high heterogeneity for FSH (*p* < 0.00001, *I* ^2^ = 93%), LH (*p* < 0.00001, *I* ^2^ = 97%), SHBG (*p* = 0.003, *I* ^2^ = 75%), and DHEA (*p* < 0.00001, *I* ^2^ = 99%) levels, while the remaining indicators showed homogeneity using a random‐effects model. Meta‐analysis showed that six studies^22^, ^23^, ^24^, ^25^, ^28^, ^29^ reported FSH levels (MD: 0.19; 95% CI: −0.29, 0.68; *p* = 0.44) (Figure 6A), seven studies^21^, ^22^, ^23^, ^24^, ^25^, ^28^, ^29^ reported LH levels (MD: −0.83; 95% CI: −1.01, −0.64; *p* < 0.00001) (Figure 6B), three studies^22^, ^23^, ^29^ reported E_2_ levels (MD: −3.07; 95% CI: −5.46, −0.68; *p* = 0.01) (Figure 6C), five studies^24^, ^25^, ^26^, ^28^, ^29^ reported SHBG levels (MD: −2.08; 95% CI: −5.29, 1.14; *p* = 0.21) (Figure 6D), three studies^24^, ^25^, ^28^ reported DHEA levels (MD: −0.75; 95% CI: −2.93, 1.43; *p* = 0.50) (Figure 6E), and three studies^25^, ^26^, ^28^ reported FAI values (MD: −0.99; 95% CI: −1.41, −0.56; *p* < 0.00001) (Figure 6F). Compared to the control group, the dietary group showed improvements in terms of LH levels, E_2_ levels, and FAI values. Subgroup analysis revealed that the Mediterranean diet, low‐calorie diet combined with a low‐carbohydrate diet and energy‐restricted diet groups had improved LH levels, the energy‐restricted diet group had improved E_2_ levels, and the low‐calorie diet combined with an extract diet group had improved FAI values; overweight PCOS patients and obese PCOS patients in the dietary intervention group had improved LH and E_2_ levels and LH levels, respectively. Subgroup analysis results for hormones and related indicators are shown in Table 5.

**TABLE 5:** Subgroup analyses evaluated the effects of different dietary regimens and BMI types on hormones and related indicators in overweight/obese patients with PCOS.

Subgrouped by	Number of trials	Effect size	95% CI	*p* value for effect estimates^2^	*I* ^*2*^, %	*p* value for within‐subgroup heterogeneity^2^	*p* value for between‐subgroup heterogeneity^3^
LH, IU/L							
Dietary patterns							
Mediterranean diet	1	−4.42	−6.22 to −2.62			<0.00001	0.005
Calorie‐restricted diet	1	−2.35	−2.90 to −1.80			<0.00001	
Low‐energy and low‐carbohydrate diet	1	−2.35	−2.91 to −1.79			<0.00001	
Low‐calorie combined extract diet	3	−1.45	−3.86 to 0.96			0.24	
Very‐low‐calorie diet	1	0.10	−3.68 to 3.88			0.96	
BMI types							
Overweight (≥25 kg/m^2^)	3	−2.44	−2.83 to −2.06	0.09	59	<0.00001	<0.00001
Obese (≥30 kg/m^2^)	4	−0.83	−1.01 to −0.64	<0.00001	97	0.0007	
E_2_, IU/L							
Dietary patterns							
Calorie‐restricted diet	1	−4.44	−7.72 to −1.16			0.008	0.01
Low‐energy and low‐carbohydrate diet	1	−1.60	−5.19 to 1.99			0.38	
Low‐calorie combined extract diet	1	0.00	−14.97 to 14.97			1.00	
BMI types							
Overweight (≥25 kg/m^2^)	2	−3.15	−5.57 to −0.73	0.25	24	0.01	0.01
Obese (≥30 kg/m^2^)	1	0.00	−14.97 to 14.97			1.00	
FAI, %							
Dietary patterns							
Low‐calorie combined extract diet	1	−0.96	−1.40 to −0.52			<0.0001	<0.00001
Low‐calorie, low‐carbohydrate combined extract diet	1	−1.37	−4.59 to 1.85			0.40	
Very‐low‐calorie diet	1	−1.75	−4.47 to 0.97			0.21	

#### 3.3.5 Fertility‐related indicators

**FIGURE 7:**
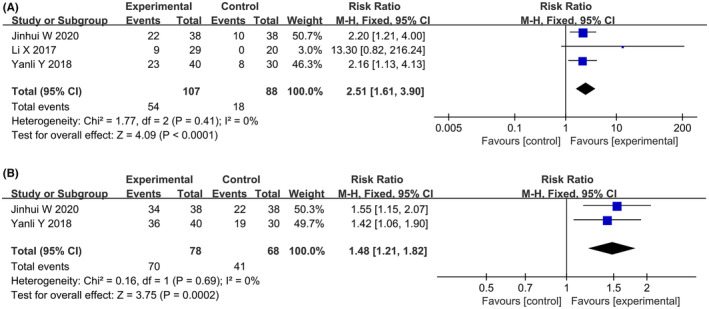
Forest plots of meta‐analysis for (A) the number of pregnancies and (B) the number of ovulations. CI, confidence interval; df, degrees of freedom; M‐H, Mantel–Haenszel.

Three studies reported the impact of dietary interventions on fertility‐related outcomes. Heterogeneity tests revealed that all the indicators were homogeneous in a fixed‐effects model. A meta‐analysis indicated that three studies^21^, ^22^, ^23^ reported the number of pregnancies (RR: 2.51; 95% CI: 1.61, 3.90; *p* < 0.0001) (Figure 7A), and two studies^22^, ^23^ reported the number of ovulations (RR: 1.48; 95% CI: 1.21, 1.82; *p* = 0.0002) (Figure 7B). Compared to the control group, the dietary intervention group showed more advantages in terms of the number of pregnancies and ovulations. Subgroup analysis revealed that a limited energy diet and a low‐calorie diet combined with a low‐carbohydrate diet had advantages in terms of pregnancy and ovulation. Subgroup analysis results for fertility‐related indicators are shown in Table 6.

**TABLE 6:** Subgroup analyses evaluated the effects of different dietary regimen and BMI types on fertility‐related indicators in overweight/obese patients with PCOS.

Subgrouped by	Number of trials	Effect size	95% CI	*p* value for effect estimates^2^	*I* ^*2*^, %	*p* value for within‐subgroup heterogeneity^2^	*p* value for between‐subgroup heterogeneity^3^
The number of pregnancies, *n*							
Dietary patterns							
Mediterranean diet	1	13.30	0.82 to 216.24			0.07	<0.0001
Calorie‐restricted diet	1	2.20	1.21 to 4.00			0.01	
Low‐energy and low‐carbohydrate diet	1	2.16	1.61 to 3.90			0.02	
The number of ovulations, *n*							
Dietary patterns							
Calorie‐restricted diet	1	1.55	1.15 to 2.07			0.004	0.0002
Low‐energy and low‐carbohydrate diet	1	1.42	1.06 to 1.90			0.02	

## 4 DISCUSSION

For the first time, this systematic review and meta‐analysis evaluated the effects of dietary interventions on overweight and obese patients (BMI ≥ 25 kg/m^2^) with PCOS. The aim was to analyze the effectiveness of dietary interventions for improving body composition, glucose and lipid metabolism, endocrine levels, and pregnancy outcomes in PCOS patients. In the following section, we mainly address five dietary approaches. First, the Mediterranean diet is primarily based on the consumption of vegetables, fruits, fish, whole grains, legumes, and olive oil, emphasizing the freshness, seasonality, and simple processing of food,^30^ which helps reduce the risk of various chronic diseases and promote longevity. Second, a low‐calorie diet refers to a daily calorie intake below normal levels, usually set at around 3347–5021 kJ (800–1200 kcal),^31^ mainly used for weight loss or weight management, requiring adequate nutrients to avoid potential health risks. Third, a low‐carbohydrate diet, meaning daily carbohydrate intake accounted for less than 20% of the total calories. Meanwhile, fat (55%–65% of daily calories) and protein (25–30% of daily calories) intake proportions are increased to reduce insulin secretion and glycogen storage,^32^ resulting in weight loss. Fourth, an energy‐restricted balanced diet ensures sufficient intake of vitamins, minerals, dietary fiber, and water while controlling daily total energy intake and has two common forms: reducing 30%–50% or 500–1000 kcal from the target energy, and limiting total energy to 1200–1400 kcal for men and 1000–1200 kcal for women, but not less than 1000 kcal per day. Fifth, the effectiveness of extract‐based diets is related to the source of the extracts. For example, the cardamom extract mentioned in this paper is rich in isoflavones and flavonoids and effectively reduces fat tissue storage,^33^ while thylakoid‐rich spinach extract enhances insulin sensitivity, alleviates insulin resistance, lowers blood sugar levels, improves glucose homeostasis, and enhances metabolism to achieve weight loss.^25^ In conclusion, these five dietary approaches have their own characteristics and should be chosen according to the individual needs of patients.

The meta‐analysis results showed that compared to the control interventions, dietary interventions effectively improved the following: (1) Weight‐related indicators, with reductions in weight, BMI, WC, HC, and the WHR; (2) glucose metabolism, with reductions in FBG, FINS, and HOMA‐IR levels; (3) lipid metabolism, with reductions in LDL‐C and TC levels; (4) endocrine indicators, with reductions in LH levels, E_2_ levels, and FAI values; and (5) pregnancy outcomes, with increases in the number of pregnancies and ovulations. Additionally, dietary interventions did not significantly affect HDL‐C, TG, FSH, SHBG, and DHEA levels. Subgroup analysis showed that among the different dietary interventions, energy‐restricted diets, low‐calorie combined with low‐carbohydrate diets, and low‐calorie combined with extract diets improved metabolic function in PCOS patients with a BMI ≥ 25 kg/m^2^; in particular, energy restricted diets also improved pregnancy outcomes, and these diets should be recommended as clinical dietary plans. A Mediterranean diet can improve LH levels while promoting weight loss. Although very low‐calorie diet does not lead to weight loss, it has advantages in improving fasting blood glucose. Low‐carbohydrate combined with extract diet and low‐calorie low‐carbohydrate combined with extract diet do not have advantages in all aspects. In terms of BMI type, dietary intervention can effectively reduce weight in both overweight and obese stages, but intervention in the overweight stage can improve glucose and lipid metabolism as well as a measure of endocrine activity with a more comprehensive impact than intervention in the obese stage.

In the international context, dietary interventions have been widely recommended as a first‐line treatment for PCOS and are particularly applicable to overweight and obese patients. Research suggests that the clinical manifestations of PCOS change with increasing body weight, leading to insulin resistance and a range of symptoms related to hyperandrogenism, ultimately resulting in adverse pregnancy outcomes.^34^ Weight gain is associated with worsened insulin sensitivity, possibly due to inflammation or intestinal dysbiosis.^35^ Insulin resistance plays a significant role in the development of metabolic dysfunction, primarily causing abnormalities in blood glucose and lipids and hypertension. Elevated insulin levels also promote excessive ovarian androgen secretion and decrease the synthesis of SHBG in the liver, further exacerbating hyperandrogenism. Conversely, hyperandrogenism is positively correlated with glucose metabolism abnormalities and is an independent risk factor for metabolic dysfunction.^36^ Extensive clinical practice has demonstrated that dietary interventions can effectively reduce weight, improve insulin resistance and hyperandrogenism, and improve pregnancy outcomes.^37^, ^38^ It is worth noting that different dietary interventions have different calorie ranges, and low‐calorie diets have more significant effects. Several studies have found that energy‐restricted diets can improve cardiovascular and reproductive parameters by mediating changes in insulin resistance after weight loss,^39^ while short‐term, low‐calorie diets can increase SHBG levels, effectively reducing serum insulin levels, and improving menstrual and reproductive outcomes.^40^ Among these interventions, although energy‐restricted diets fall under the category of low‐calorie diets, their nutritional structure is closer to a balanced diet within the restricted energy range and leans toward an independent dietary approach. Therefore, the following analysis was conducted to independently evaluate this dietary intervention as well as the other two types of low‐calorie diets.

According to our meta‐analysis results, among the three recommended dietary plans, energy‐restricted diets should be the preferred option, as their effectiveness in weight reduction has been confirmed by relevant meta‐studies.^41^ These diets can also improve glucose and lipid metabolism, endocrine levels, and pregnancy outcomes, thereby reducing the risk of type 2 diabetes and cardiovascular metabolic diseases, which is consistent with the conclusions of several recent studies.^42^, ^43^, ^44^, ^45^ Compared to the other two diet types, energy‐restricted diets can effectively reduce the levels of TC and LDL‐C, which may be related to the proportion of soy protein intake, as found in previous studies.^46^ There is ample evidence supporting the effectiveness of energy‐restricted diets in improving insulin levels in overweight or obese patients with PCOS. In particular, we found that the intervention effect of dietary restriction on overweight is better, which is consistent with the conclusion that early energy‐restricted diet intervention is more effective and has positive effects on weight, endocrine characteristics, cardiovascular metabolic risk status, and pregnancy outcomes proposed by previous studies.^47^, ^48^ However, there is no advantage of restricting energy intake for improving hyperandrogenemia in Kaohsiung, which contradicts the conclusion of the previous related meta‐study that this dietary approach plays a key role in FAI values.^20^ Considering the possible differences in diagnostic criteria and the inclusion of only the 2003 Rotterdam criteria in this study, further high‐quality clinical trials are needed to verify the effectiveness of FAI improvement. The combination of low‐calorie and low‐carbohydrate or extract diets differ in their impact due to different combination methods. The common aspect is that both methods have advantages in regulating FGB and FINS levels. The difference is that low‐calorie combined with low‐carbohydrate diets can directly reduce BMI values and insulin levels, and can also improve pregnancy outcomes; low‐calorie combined with extract diets cannot reduce BMI values but can reduce body weight, WC, HC, and WHR values, effectively playing a role in weight reduction and shaping, especially in reducing FAI values and improving hyperandrogenemia, which is the biggest difference from other dietary approaches. However, as insulin resistance is the most critical pathogenic feature of PCOS, low‐calorie combined with low‐carbohydrate diets that can improve HOMA‐IR values should be recommended as the second dietary approach, while low‐calorie combined with extract diets are more suitable for PCOS patients who need weight loss and shaping, have hyperandrogenemia characteristics, and have low fertility requirements. Combining the analysis results of other dietary approaches, neither low‐carbohydrate combined with extract diets nor low‐calorie, low‐carbohydrate combined with extract diets have advantages. It can be considered that low‐calorie diets play a major role in the two combined dietary approaches, suggesting that dietary structures with increasing complexity are not necessarily more effective and should be selected based on the clinical characteristics of PCOS. The above‐described results expand upon the existing research, which reports the effectiveness of three types of low‐calorie dietary approaches, among which the restriction of energy intake as a more balanced dietary structure is more conducive to long‐term and effective implementation in many PCOS patients, improving patient compliance and effectively improving various indicators.

Considering the issue of significant clinical heterogeneity, this study only included research that met both the 2003 Rotterdam diagnostic criteria and the RCT requirement to minimize bias caused by diagnostic differences and nonstandardized procedures. However, after specific analysis, it was found that there were still some indicators with high heterogeneity. In order to further explore the source of heterogeneity, this study conducted subgroup analysis and sensitivity analysis on indicators that showed significant significance. The comprehensive analysis suggested that the heterogeneity in BMI was derived from the low‐calorie combined extract diet (after excluding the study by Cheshmeh S (2020), *I* ^2^ = 41%). According to previous research results, the extract used in this study was cardamom, which has anti‐inflammatory effects,^49^ is related to the PCOS inflammatory response, and can synergistically improve metabolic syndrome with dietary methods. Cardamom may significantly increase BMI,^50^, ^51^ but it can also be used to treat muscle‐deficient obesity.^52^ This may be the reason why low‐calorie combined extract diets do not have advantages in improving BMI but can improve other indicators such as weight, WC, and HC. In addition, the study included a larger number of people and a wider range of BMIs than other studies, which may be the reason for the higher sensitivity. The heterogeneity in LH levels was derived from the studies on low‐calorie‐extract combined diets (after excluding the study by Tabrizi F (2020), *I* ^2^ = 27%). There were fewer studies related to spinach‐derived liposomes included in this study, and some studies have shown that spinach‐derived liposomes combined with high‐intensity functional training can effectively reduce insulin resistance levels in obese males.^53^ Based on the subjects and combined methods, it is possible that this extract is not significantly advantageous for insulin resistance, which may be the reason for the higher sensitivity. A specific analysis of the source of heterogeneity in the WHR, FBG level, and FINS levels could not be conducted due to the limited number of studies on each type of diet. Therefore, although low‐calorie combined with extract diets have advantages for improving obesity and clinical characteristics, the impact of this diet combination on weight and endocrine levels still needs to be verified in additional studies due to the high heterogeneity in BMI and LH levels, which are the main indicators.

## 5 STRENGTHS AND LIMITATIONS

Our study had the following important advantages: (1) the strict inclusion of RCTs based on the 2003 Rotterdam diagnostic criteria; (2) the inclusion of participants with clear characteristics based on various indicators: (a) participants aged 18–45 years; (b) participants who met the international standard BMI ≥25 kg/m^2^; and (c) women with PCOS; and (3) the strict selection of intervention methods primarily focused on dietary interventions. These points are beneficial for reducing bias in the discussion process and focusing on the impact of dietary interventions on the weight and clinical manifestations of PCOS patients with a BMI ≥25 kg/m^2^, especially on reproductive outcomes. This study supplements the previous lack of evidence‐based content in various meta‐analyses. We also conducted subgroup analysis based on different dietary patterns and BMIs, explored the suitable population for different types of diets and the timing of intervention treatment, and provided effective dietary recommendations for obese PCOS patients, facilitating long‐term patient adherence. The limitations of this analysis lie in the strict inclusion criteria, resulting in incomplete inclusion of dietary types and fewer studies on various types of diets. This limitation is also an important factor that contributed to bias in the analysis, which could not be avoided. Furthermore, most diets have limited safety evaluations, making it difficult for us to make effective judgments on safety, further limiting the analysis. This finding indicates that the efficacy of dietary interventions requires a large number of high‐quality clinical trials for verification with a focus on safety to better assess the characteristics of different dietary patterns.

## 6 CONCLUSIONS

Our meta‐analysis, which focused on the impact of diet on PCOS patients, validated and supplemented previous evidence‐based findings. Compared to previous conclusions, our meta‐analysis specifically examined the effect of dietary patterns on PCOS patients using a BMI ≥25 kg/m^2^ as the criterion. This allowed us to accurately and specifically study the effectiveness of dietary patterns for the majority of PCOS patients. Among the three recommended dietary patterns for overweight or obese PCOS patients, namely energy‐restricted diets, low‐calorie combined with low‐carbohydrate diets, and low‐calorie combined with extract diets, all three types of diets were found to effectively improve clinical manifestations and pregnancy outcomes based on weight loss. However, energy‐restricted diets are considered the primary recommendation in clinical practice due to their wider applicability for PCOS patients. These diets not only restrict energy intake but also ensure that nutritional requirements are met, with macronutrient proportions in line with a balanced diet. Furthermore, these diets are easier for patients to adhere to in the long term. Low‐calorie combined with low‐carbohydrate diets and low‐calorie combined with extract diets are recommended as secondary and tertiary options, respectively. It is worth noting that other types of diets included in our analysis showed either limited advantages or no advantages. This may be due to incomplete data, the small number of studies, or regional disparities, resulting in biased conclusions compared to various clinical trials. Therefore, further high‐quality RCTs are needed to validate the advantages of the Mediterranean diet, very low‐calorie diets, and low‐carbohydrate diets. Subsequent clinical trials should provide specific descriptions of dietary structures, compliance, and safety for more accurate dietary plans for overweight and obese PCOS patients.

## FUNDING INFORMATION

This study was supported by the Natural Science Foundation Program of Fujian Provincial Science and Technology Department (2023J011231) and the Science and Technology Innovation Startup Fund of Fujian Maternity and Child Health Hospital, the introduction of talent supporting scientific research projects (No. Maternal and Child YCXY 24‐01) and the Startup Fund for scientific research, Fujian Medical University(2023QH1185).

## CONFLICT OF INTEREST STATEMENT

The authors declare no conflict of interest.

## Data Availability

The original contributions presented in the study are included in the article/Supplementary Material. Further inquiries can be directed to the corresponding author.
